# Effect of increasing age on percutaneous coronary intervention vs coronary artery bypass grafting in older adults with unprotected left main coronary artery disease: A meta‐analysis and meta‐regression

**DOI:** 10.1002/clc.23253

**Published:** 2019-09-05

**Authors:** Mahin R. Khan, Waleed T. Kayani, Waqas Ahmad, Malalai Manan, Ravi S. Hira, Ihab Hamzeh, Hani Jneid, Salim S. Virani, Neal Kleiman, Nasser Lakkis, Mahboob Alam

**Affiliations:** ^1^ Division of Cardiology McLaren‐Flint/Michigan State University Flint Michigan; ^2^ Section of Cardiology, Department of Internal Medicine Baylor College of Medicine Houston Texas; ^3^ Department of Internal Medicine Nishtar Medical University Multan Pakistan; ^4^ Department of Internal Medicine King Edward Medical University Lahore Pakistan; ^5^ Division of Cardiology University of Washington Seattle Washington; ^6^ Section of Cardiology Michael E. DeBakey Veterans Affairs Medical Center Houston Texas; ^7^ Houston Methodist DeBakey Heart and Vascular Center Houston Methodist Hospital Houston Texas

**Keywords:** coronary artery bypass graft surgery, elderly, percutaneous coronary intervention, unprotected left main coronary artery

## Abstract

**Background:**

Older adults (≥70‐year‐old) are under‐represented in the published data pertaining to unprotected left main coronary artery disease (ULMCAD). Hypothesis: Percutaneous coronary intervention (PCI) might be comparable to coronary artery bypass grafting (CABG) for revascularization of ULMCAD.

**Methods:**

We compared PCI versus CABG in older adults with ULMCAD with an aggregate data meta‐analyses (4880 patients) of clinical outcomes [all‐cause mortality, myocardial infarction (MI), repeat revascularization, stroke and major adverse cardiac and cerebrovascular events(MACCE)] at 30 days, 12‐24 months & ≥36 months in patients with mean age ≥70 years and ULMCAD. A meta‐regression analysis evaluated the effect of age on mortality after PCI. Odds ratios (OR) and 95% confidence intervals (CI) were estimated using random‐effects model.

**Results:**

All‐cause mortality between PCI and CABG was comparable at 30‐days (OR0.77, 95% CI 0.42‐ 1.41) and 12‐24‐months (OR 1.22, 95% CI 0.78‐1.93). PCI was associated with a markedly lower rate of stroke at 30‐day follow‐up in octogenarians (OR 0.14, 95% CI 0.02‐0.76) but an overall higher rate of repeat revascularization. At ≥36‐months, MACCE (OR 1.26,95% CI 0.99‐1.60) and all‐cause mortality (OR 1.39, 95% CI 1.00‐1.93) showed a trend favoring CABG but did not reach statistical significance. On meta‐regression, PCI was associated with a higher mortality with advancing age (coefficient=0.1033, p=0.042).

**Conclusions:**

PCI was associated with a markedly lower rate of early stroke in octogenarians as compared to CABG. All‐cause mortality was comparable between the two arms with a trend favoring CABG at ≥36‐months.PCI was however associated with increasing mortality with advancing age as compared to CABG.

## 1 INTRODUCTION

Supportive evidence from randomized controlled trials^1^, ^2^, ^3^ and meta‐analyses^4^, ^5^ has substantiated the use of percutaneous coronary intervention (PCI) as an alternate to coronary artery bypass grafting (CABG) for revascularization of unprotected left main coronary artery (ULMCA) disease in patients with anatomy amenable to PCI.^1^, ^6^ These findings are reflected in the 2011 American Heart Association/Society of Coronary Angiography and Intervention/ American College of Cardiology Foundation (AHA/SCAI/ACCF)^7^ and 2014 European Guidelines^6^ on myocardial revascularization. In terms of complications, CABG is associated with a higher peri‐procedural mortality and stroke while PCI is associated with a higher rate of repeat revascularization.^5^, ^8^ Older adults defined as age ≥70 years are generally under‐represented in studies and are a cohort with greater frailty, more comorbidities and higher procedural complication rates.^9^, ^10^ Existing data is limited by smaller sample sizes which makes it challenging to draw robust conclusions regarding true efficacy and risks of each therapy. Our study attempts to explore the optimal revascularization technique for ULMCA disease in the elderly by evaluating all existing data.

## 2 METHODS

**Figure 1:**
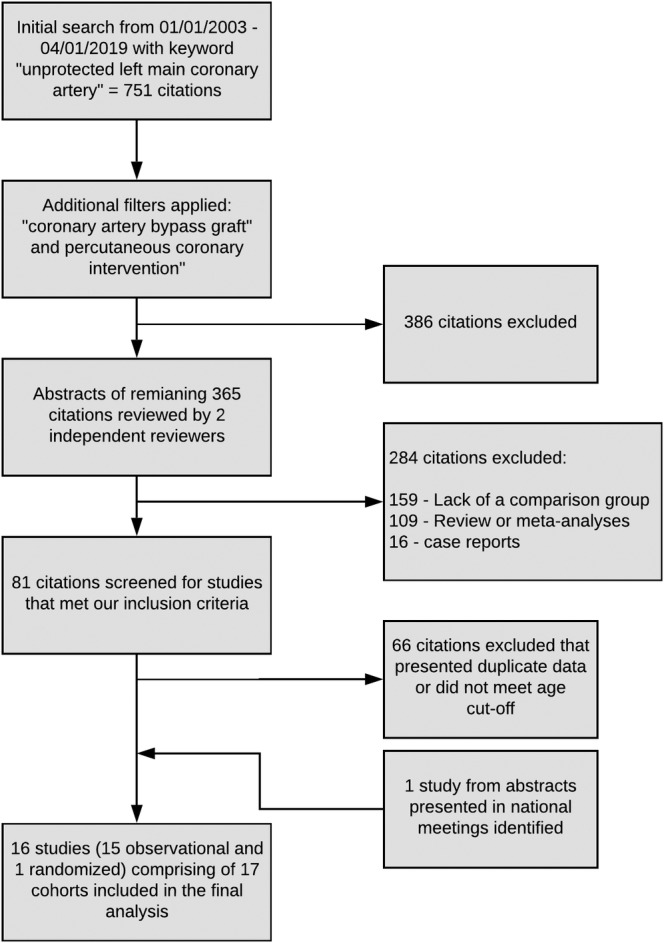
Data search and review method—Flowsheet of identification of the studies that were included in the analyses

A systematic data search was performed using keywords “unprotected left main coronary artery, coronary artery bypass graft” and PCI from January 1, 2003 to April 1, 2019 on MEDLINE. The initial search resulted in 365 citations, a careful review of the abstracts of these citations identified 81 studies that reported comparative outcomes of PCI vs CABG in ULMCA lesions. Sixty six of these identified citations were excluded either because of duplicated data (population reported elsewhere) or if they did not meet our age cutoff (mean age ≥70 years in the PCI arm). The study by Gomez et al^11^ compared PCI with CABG in 3 patient cohorts, out of which 2 were in included in our analysis as they met our age cutoff. Finally, abstracts presented in national cardiovascular conferences were searched to identify studies meeting our inclusion criteria (Figure 1). In the final analysis, **16 studies** (15 observational and 1 randomized) comprising of 17 patient cohorts were included (supplementary table 1).

Each of these studies were reviewed and data were extracted independently by two reviewers (Mahin R. Khan and Waleed T. Kayani). Data pertaining to baseline demographic and clinical variables including clinical presentation, risk stratification scores, coronary anatomy, and procedural variables were obtained. The initial revascularization strategy (CABG or PCI) was the primary independent variable. The primary outcome of our study was all‐cause mortality, secondary outcomes included nonfatal myocardial infarction (MI), stroke, repeat revascularization, and major adverse cardiac and cerebrovascular events (MACCE; composite end‐point of all‐cause mortality, MI, stroke, or repeat revascularization). The outcomes are reported at a 30‐day, 12‐24 months and ≥36 months follow‐up in a cumulative fashion. The studies' defined end points were used to conduct our meta‐analyses. Additional comparative analyses performed included analyses to compute inter‐group differences between the ≥70‐year‐old and ≥80‐year‐old patients. The funnel plot of all‐cause mortality shows a symmetrical distribution of studies indicating that a publication bias is less likely (supplementary figure 1). The distributions of continuous and categorical variables were described using mean SD and percentages, respectively. Continuous variables were compared using the 2‐tailed student *t*‐test while the categorical variables were compared using chi‐square test with Yates' correction, where applicable. Odds ratios (ORs) and their 95% confidence intervals (CIs) were used to summarize the effect of each outcome at the corresponding follow‐up using the random‐effects model. Cochran's Q‐statistic and I^2^ index tests were computed to determine the heterogeneity. An I^2^ of 25% was considered to indicate statistically significant heterogeneity. We reported the results using random‐effects modeling only, given the inherent heterogeneity of the data. *P*‐values of <.05 were considered statistically significant. Furthermore, a Newcastle‐Ottawa scale was employed to assess the quality of all included observational studies. We defined the high‐quality studies as those that scored nine stars (maximum) on the scale, studies with moderate quality were defined as those that scored 78 stars. Nine of the included studies scored the maximum of nine stars while all of the remaining observational studies scored at least seven stars on the Newcastle‐Ottawa scale (supplementary Table 2).

**Figure 2:**
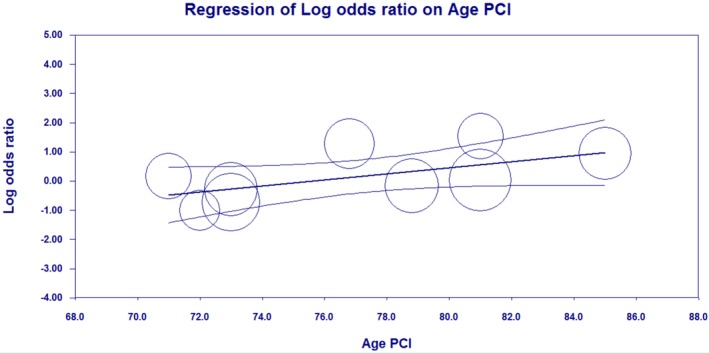
Meta regression scatterplot of mortality and age with percutaneous coronary intervention—Positive slope indicative of increasing mortality with advancing age in PCI. Circles represent individual studies included. Coefficient = 0.1033, 95% confidence interval 0.0039‐0.2027, SE = 0.0507. Z Value = 2.04, (*P*‐value = 0.0416)

We also performed meta‐regression analyses (on 10 observational studies that reported mortality at 12‐24 months) to evaluate whether the effect of PCI on mortality was modulated by age. The meta‐regression graphs are plotted as log odds ratio of mortality on the *y*‐axis against mean age as a covariate on the *x*‐axis. The meta‐regression coefficients show the increase in log OR per unit increase in age (covariate). Figure 2 describes the effect of PCI on mortality (plotted as log OR on the y‐axis) as a function of age in the PCI arm (*x*‐axis), using patients in the respective CABG group as control. The baseline data was analyzed using Statistical Package for Social Sciences, version 19.0 (IBM, Armonk, New York). The meta‐analyses were performed using Review Manager, version 5.0 (The Nordic Cochrane Center, The Cochrane Collaboration 2012, Copenhagen, Denmark). Meta‐regression analysis was performed using Comprehensive Meta‐Analysis, version 3.0 (Biostat, Englewood, New Jersey).

## 3 RESULTS

The current meta‐analyses included a total of 16 studies (15 observational and 1 randomized) and 4880 patients. Of the included studies, the primary outcome, that is, all‐cause mortality was reported in 6 studies at 30 days and ≥36 months while 10 studies reported all‐cause mortality at 12‐24 months. There were several differences in the baseline clinical characteristics of the patients undergoing PCI vs CABG (Table 1). The patients in the PCI group had a greater prevalence of chronic renal insufficiency and congestive heart failure and were more likely to present with non‐ST‐elevation myocardial infarction than patients in the CABG group (54.8% vs 50.3%). Patients in the CABG group were more likely to be men and had a higher prevalence of hyperlipidemia and smoking. Patients in the PCI group had a higher prevalence of non‐distal (ostial and mid‐shaft lesions) left main coronary artery (LMCA) stenosis but a similar frequency of distal LMCA disease as compared to the CABG group. A greater percentage of patients with LMCA with single and double‐vessel disease underwent PCI and a greater proportion of patients with LMCA with triple‐vessel disease underwent CABG. The two groups did not have any statistically significant difference in terms of age, hypertension, diabetes, mean SYNTAX scores, and prevalence of peripheral arterial disease. It is however noteworthy that SYNTAX scores were not reported for the PCI and CABG groups in 11 out of the 17 cohorts included in our analysis. Notably, 220 (10.9%) of the patients underwent bare‐metal stent implantation.

**Table 1:** Baseline and demographic variables

	PCI		CABG		
Variable	Patients (n)	Value	Patients (n)	Value	*P*‐value
Total patients	2022		2858		NA
Mean age (yrs)	1764	75.5 ± 5.1	2495	72.1 ± 5.6	.22
Men	1732	1719 (68.1%)	2446	1817 (74.3%)	<.001
Mean LVEF (%)	1231	50.9 ± 3.14	1660	49.5 ± 2.9	.33
Stable angina	773	231 (29.9%)	774	285 (36.8%)	.012
NSTEMI	1104	605 (54.8%)	1310	659 (50.3%)	.03
STEMI	366	18 (4.9%)	180	12 (6.7%)	.43
SYNTAX score	820	28.5 ± 6.3	1072	31.8 ± 3.9	.16
Diabetes mellitus	1764	626 (35.5%)	2495	871 (34.9%)	.72
Hypertension	1764	1359 (77.0%)	2495	1880 (75.4%)	.22
Hyperlipidemia^a^	1351	767 (56.8%)	1617	1021 (63.1%)	<.001
Smoker	1725	555 (32.2%)	2458	820 (33.4%)	.43
Previous MI	1007	262 (26.0%)	1455	371 (25.5%)	.78
Previous PCI	910	199 (21.9%)	1139	127 (11.3%)	.001
Chronic renal insufficiency	1404	186 (13.2%)	1888	195 (10.3%)	.01
Previous stroke	971	112 (11.5%)	1474	147 (10.0%)	.23
Previous CHF	669	192 (28.7%)	1001	247 (24.7%)	.07
Peripheral arterial disease	1143	226 (19.8%)	1639	317 (19.3%)	.81
Distal LMCAD	1336	908 (68.0%)	1123	789 (70.2%)	.24
Non‐distal LMCAD	910	247 (27.1%)	767	169 (22.1%)	.02
Isolated LMCAD	1176	96 (8.2%)	1543	80 (5.2%)	.002
LMCA with single‐vessel CAD	1240	363 (29.3%)	1605	204 (12.7%)	<.001
LMCA with two‐vessel CAD	1240	384 (31.0%)	1605	392 (24.4%)	<.001
LMCA with triple‐vessel CAD	1338	463 (34.6%)	1766	1048 (59.3%)	<.001

*Note*: Data are presented as mean ± SD or n (%).

Abbreviations: CABG, coronary artery bypass graft; CAD, coronary artery disease; CHF, congestive heart failure; LMCA, left main coronary artery; LMCAD, left main coronary artery disease; LVEF, left ventricular ejection fraction; MI, myocardial infarction; NSTEMI, non‐ST‐segment elevation MI; PAD, peripheral artery disease; PCI, percutaneous coronary intervention; STEMI, ST‐segment elevation MI.

a As defined in individual studies.

**Figure 3:**
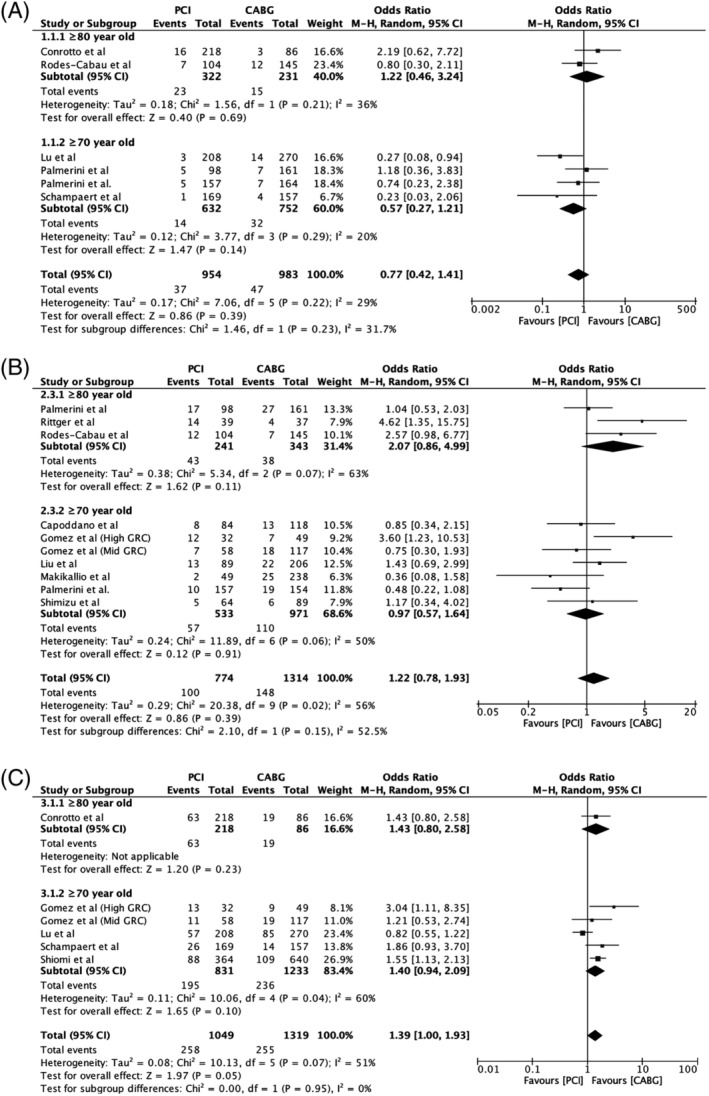
Forest plot of all‐cause mortality at (A) 30 days, (B) 12‐24 months and (C) ≥36 months follow‐up—Comparable mortality at early and intermediate follow‐ups with a trend favoring coronary artery bypass grafting at long term follow‐up

There were no intergroup differences between PCI and CABG for the primary outcome of all‐cause mortality at 30 days (OR 0.77, 95% CI 0.42‐1.41) and 12‐24 months (OR 1.22, 95% CI 0.78‐1.93). At ≥36 months, mortality rate showed a trend favoring the CABG group (19.3%) as compared to the PCI group (24.6%) but did not reach statistical significance (OR 1.39, 95% CI 1.00‐1.93) (Figure 3). At 30 days post‐intervention, the PCI and CABG groups had no intergroup differences in nonfatal MI (OR 0.84, 95% CI 0.53‐1.32) and repeat revascularization (OR 0.66, 95% CI 0.22‐2.01). Collective 30‐day MACCE (OR 0.60, 95% CI 0.35‐1.04) and stroke (OR 0.31, 95% CI 0.09‐1.02) showed a statistically insignificant trend inclining in favor of PCI. However, PCI had a marked advantage over CABG in 30‐day stroke rates in the octogenarian sub‐group (OR 0.14, 95% CI 0.02‐0.76) (supplementary figure 2). Between 12 and 24 months, PCI and CABG groups were associated with comparable rates of MACCE (OR 1.41, 95% CI 0.80‐2.48), stroke, (OR 0.33, 95% CI 0.05‐1.98) and nonfatal MI (OR 1.44, 95% CI 0.88‐2.34) but there was a higher incidence of repeat revascularization in the PCI group (OR 4.02, 95% CI 2.54‐6.36). Inter‐group differences between 70 to 79‐year‐old patients and ≥80‐year‐old patients showed a statistically significant increased rate of repeat revascularization in the ≥80‐year‐old group (*P* = 0.03) at 12‐24 months. At a follow‐up of ≥36 months, PCI and CABG had no difference in terms of incidence of stroke (OR 0.84, 95% CI 0.52‐1.34), with a greater rate of nonfatal MI (OR 1.58, 95% CI 1.10‐2.28) and repeat revascularization (OR 2.98, 95% CI 2.00‐4.44) occurring with PCI as compared to CABG. MACCE, although not significantly different with PCI (35.9%) compared to CABG (30.6%), showed an inclination towards the CABG arm (OR 1.26, 95% CI 0.99‐1.60) (Table 2).

**Table 2:** Meta‐analyses outcomes

			Event rate		OR (95% CI)			
Outcomes	Studies (N)^a^	Patients (n)^b^	PCI	CABG	Overall	70–79 years	≥80 years	Intergroup difference *P*‐value
All‐cause mortality								
30 days	6	1937	37/954	47/983	0.77 (0.42–1.41)	0.57 (0.27‐1.21)	1.22 (0.46‐3.24)	.23
12‐24 months	10	2088	100/774	138/1314	1.22 (0.78‐1.93)	0.97 (0.57‐1.64)	2.97 (0.86‐4.99)	.15
≥36 months	6	2368	258/1049	255/1319	1.39 (1.00‐1.93)	1.40 (0.94‐2.09)	1.43 (0.80‐2.58)	.95
MACCE^c^								
30 days	4	1357	60/699	83/658	0.60 (0.35–1.04)	0.40 (0.20‐0.78)	0.82 (0.39‐1.73)	.15
12‐24 months	9	1750	126/655	159/1095	1.41 (0.80‐2.48)	1.20 (0.67‐2.16)	2.55 (0.69‐9.41)	.60
≥36 months	5	1364	246/685	208/679	1.26 (0.99‐1.60)	1.26 (0.97‐1.65)	1.25 (0.74‐2.12)	.97
Revascularization								
30 days	5	1575	5/743	9/832	0.66 (0.22‐2.01)	0.83 (0.23‐2.99)	0.34 (0.04‐3.11)	.50
12‐24 months	8	1701	95/673	39/1028	3.75 (2.43‐5.78)	2.71 (1.58‐4.64)	7.04 (3.66‐13.6)	.03
≥36 months	6	2368	244/1049	134/1319	2.98 (2.00‐4.44)	2.92 (2.00‐4.44)	3.11 (0.90‐10.7)	.93
Nonfatal MI								
30 days	6	1937	42/954	48/983	0.84 (0.53–1.32)	1.02 (0.50‐2.09)	0.74 (0.41‐1.32)	.49
12‐24 months	8	1725	37/686	41/1039	1.44 (0.88‐2.34)	1.22 (0.67‐2.23)	2.24 (0.41‐12.4)	.51
≥36 months	6	2368	71/1049	58/1319	1.58 (1.10‐2.28)	1.60 (1.09‐2.35)	1.33 (0.36‐3.08)	.79
Stroke								
30 days	4	1129	4/520	18/609	0.31 (0.09‐1.02)	0.64 (0.09‐4.51)	0.14 (0.02‐0.76)	.24
12–24 months	4	604	11/271	27/333	0.33 (0.05–1.98)	0.29 (0.05‐1.79)	0.30 (0.01‐9.62)	.99
≥36 months	4	2112	46/959	69/1153	0.84 (0.52–1.34)	0.81 (0.43‐1.52)	0.68 (0.19‐2.38)	.81

Abbreviations: CABG, coronary artery bypass grafting; CI, confidence interval; I^2^, index for degree of heterogeneity; MACCE, major adverse cardiac and cerebrovascular events; MI, myocardial infarction; OR, odd's ratio; PCI, percutaneous coronary intervention; Q, Cochran's Q‐score for heterogeneity.

a Studies reporting the outcome.

b Number of patients included in the analysis.

c Composite endpoint of death, nonfatal MI, stroke, and repeat revascularization.

On the meta‐regression analyses, at 12‐24 months, there was a statistically significant increase in mortality in the PCI group with advancing age (71 ± 7‐85 ± 3 years) in the PCI arm (coefficient = 0.1033, 95% CI 0.004‐0.203, *P* = 0.0416) as shown in Figure 2.

## 4 DISCUSSION

In our analysis of 4880 patients with ULMCA disease and a mean age ≥70 years, PCI was associated with lower rates of stroke (in octogenarians at 30 days), higher rates of repeat revascularization (≥12 and ≥36 months) and nonfatal MI as compared to CABG. Although there was no difference in MACCE and mortality between the two groups at short (30 days) and intermediate‐term follow up (12‐24 months), long‐term follow up (≥36 months) showed a trend favoring CABG that did not reach statistical significance. The initial advantage of PCI in the incidence of stroke became nonsignificant at long‐term follow‐up, however, the disparity in repeat revascularization persisted. This observation is in conformity with prior studies including randomized controlled trials^1^, ^2^, ^12^, ^13^, ^14^, ^15^ and registry data^16^, ^17^, ^18^ in younger patients.

Results from the SYNTAX,^3^ PRECOMBAT,^19^ EXCEL^1^ trials, and meta‐analyses^5^, ^8^ comparing PCI and CABG for unprotected LMCA disease also showed no intermediate and long‐term mortality difference between the two treatment groups. The NOBLE^2^ trial also reported a comparable mortality rate between the PCI and CABG arms but there was a potential concern of the study being low‐powered to detect outcomes due to early termination of the trial secondary to a low event rate. Despite more comorbidities, all‐cause mortality at ≥36 months in our analyses was comparable in the PCI (24.6%) and CABG (19.3%) arms (OR 1.39, 95% CI 1.00‐1.93; *P* = 0.05), that is, preferring CABG but not reaching statistical significance. A subgroup analysis of ≥80‐year‐old patients in our study revealed no significant inter‐group differences compared to the ≥70‐year‐old patients (Table 2). In the EXCEL trial,^20^ patients >75‐year‐old patients had an all‐cause mortality of 16.6% in the PCI arm and 8.4% in the CABG arm (OR 1.96, 95% CI 1.00‐3.83), with no inter‐group difference between ≤75 and >75‐year‐old patients (*P* = 0.21). With meta‐regression analyses, we observed that advancing age was associated with a higher mortality with PCI (Figure 2). Since all of the studies included in the regression analyses were observational, there is a high likelihood of referral bias, where older patients with more comorbidities preferentially underwent PCI instead of CABG.

There was a marked advantage of PCI over CABG in terms of stroke at a 30‐day follow‐up in the octogenarian population (OR = 0.14), however the advantage was not seen at 12‐24 months and ≥36 months follow‐up. This trend is in conformity with the NOBLE^2^ trial. The elderly sub‐group of the EXCEL^20^ trial, however, did not detect statistically significant difference in the stroke rates in the both the treatment arms at short and long‐term follow‐ups, which might be secondary to a low event rate at 30 days in the PCI and CABG groups, that is, 1.3% and 0.6% respectively. The incidence of stroke at ≥36 months in our analysis was 4.8% (PCI) and 6.0% (CABG) which was similar to the stroke rate in the >75‐year sub‐group (4.9% vs 5.6%) but higher than the stroke rate in the ≤75‐year‐old subgroup, 2.2% vs 3.8%, respectively in PCI and CABG arms of the EXCEL trial.^20^ The benefit of PCI over CABG in terms of stroke, that has also been proven in a comprehensive meta‐analyses of 14 randomized controlled trials (PCI = 0.34% and CABG = 1.20% OR: 2.94, 95% CI 1.69‐5.09),^21^ is a significant finding especially in the octogenarians.

The overall rates of repeat revascularization in our analyses in patients undergoing PCI (23.3%) and CABG (10.2%) at ≥36 months was much higher than the reported rates in EXCEL^1^ (PCI, 0.7%; CABG, 1.4%), NOBLE^2^ (PCI, 16%; CABG, 10%) and elderly sub‐group of the EXCEL^1^ trial (PCI, 11.4%; CABG, 5.4%). Also, 10% of the patients in our population received bare‐metal stents. Whereas comparatively, the EXCEL^1^ trial had patients with less complex coronary anatomy (SYNTAX = 20.6 ± 6.2 in the PCI arm) and employed second generation Drug eluting stent (DES), likely leading to a lower rate of repeat revascularization. The higher rate of repeat revascularization in the NOBLE trial in the PCI group at 5 years (16%) could likely be secondary to the use of first‐generation DES in 11% of the patients and the use of Intravascular ultrasound (IVUS) in <50% of the patients,^2^ where both the advancements in PCI have led to better overall outcomes.^22^, ^23^ Significant proportion of patients undergoing PCI in our analyses underwent stenting of a distal LMCA lesion involving the bifurcation, that has been associated with increased repeat revascularization. The technique for LMCA bifurcation stenting in the included studies was also variable, ranging from single stent to “T,” crushing, and kissing stents. Data comparing individual techniques for bifurcation revascularization is scarce, however the final choice of employed technique is made considering anatomic and clinical complexity, bifurcation angle and operator experience.^24^

In our analyses, MACCE showed a trend that favored PCI at 30‐day follow‐up (OR 0.60, 95% CI 0.35‐1.04), equivalent at 12 months (OR 1.41, 95% CI 0.80‐2.48) and favoring CABG at a longer‐term follow‐up of ≥36 months (OR 1.26, 95% CI 0.99‐1.60). At long‐term follow up, the accrual difference between the two treatment modalities in terms of repeat revascularization (PCI: 23.3%, CABG: 10.2%, Number needed to harm (NNH) = 8) was significantly higher than the accrual difference in terms of stroke (PCI; 4.8%, CABG; 6.0%, Number needed to treat (NNT) = 83), which may have tilted MACCE in favor of CABG. It is however noteworthy that the “hard” outcome of stroke is practically and clinically more devastating than repeat revascularization and affects morbidity and quality of life very differently.

Our meta‐analyses have compared the outcomes of PCI and CABG in the high‐risk cohort of patients ≥70‐year‐old with a subgroup analyses of octogenarians. It is the largest report to date of all the available data comparing PCI and CABG in the elderly that includes comprehensive meta‐analyses of multiple clinical outcomes at a short, intermediate and long‐term follow‐up. We included patients from different regions of the world (supplementary table 1) with varying clinical complexities. Our analyses included data predominantly from prospective studies and propensity‐adjusted retrospective case series, which along with the use of bare metal stents in 10% of the patients, might be reflective of real‐world practices where multiple factors including risk of bleeding, non‐compliance, vessel size and other considerations guide the ultimate interventional strategy. Unifying MACCE to include repeat revascularization provides robust scientific validity to our analyses and every effort was made to standardize outcomes wherever possible.

Our analyses had limitations. All except one study included in our analyses were non‐randomized, which could have confounded the results and introduced a potential selection bias but the quality assessment of the included studies with the Newcastle‐Ottawa scale showed that 9 of the included observational studies scored 9 stars (maximum) while 7 studies scored a minimum of 7 stars (moderate quality). We did not have access to patient data. We had limited data on post‐interventional medical therapies, degree of symptomatic improvement and the effect of revascularization on patients' quality of life. As we used mean age in the PCI arm to define the age cutoff, there is a chance that 8/17 studies included some patients who might have been <70‐year‐old, however, on the subgroup analysis of ≥80‐year‐old patients, we did not see an influence of those studies on outcomes other than repeat revascularization at 12‐24 months. Our report was also limited by the absence of a longer‐term follow‐up where venous graft attrition and divergence in revascularization might become more evident. We did not have access to individual patient‐data and end‐point definitions could have been different in various studies, however we tried to carefully extract data with standardized outcomes wherever possible. It was also difficult to ascertain the complexity of underlying coronary artery disease, as most studies including the RCT did not report the SYNTAX score in the two treatment arms. Also, three included studies provided propensity‐matched outcomes with a potential for a selection bias and consequent effect on outcomes when combined with unadjusted studies. However, a previously performed subgroup analysis after exclusion of these studies did not show an effect on clinical outcomes.^25^ Similar to our meta‐analyses, the meta regression analysis was performed with the mean age of PCI and CABG arm reported in each study, there is a chance that a proportion of the included patients were <70 years of age, raising concerns about generalizability of the study to all older adults.

## 5 CONCLUSION

Older adults who undergo ULMCA revascularization had no difference in mortality with PCI or CABG at short and intermediate‐term follow‐ups. At long‐term follow‐up, CABG showed a favorable mortality trend as compared to PCI. Advancing age was also associated with increasing mortality with PCI. PCI was however associated with a markedly lower rate of early stroke in octogenarians as compared to CABG.

## CONFLICT OF INTEREST

The authors declare no potential conflict of interests.

## Supporting information


**Figure S1.** Funnel‐plot of all‐cause mortality for analysis of publication bias—Symmetric inverted funnel indicates that publication bias is less likely.
**Figure S2.** Forest plot of stroke at (A) 30‐days and (B) ≥36 months follow‐up—Decreased 30‐day stroke rate with PCI in ≥80‐year‐old patients and no statistically significant difference between PCI and CABG seen at ≥36 months.
**Table S1.** Summary of all studies included in the meta‐analyses.
**Table S2.** Newcastle‐Ottawa Scale to assess the quality of each selected study.
