# Supplementary material for: Effect of increasing age on percutaneous coronary intervention vs coronary artery bypass grafting in older adults with unprotected left main coronary artery disease: A meta‐analysis and meta‐regression
Source: Clin Cardiol. 2019 Sep 5;42(11):1071–8. doi: 10.1002/clc.23253 (PMC6837018; doi:10.1002/clc.23253)

**Supplementary Table 1. Summary of all studies included in the meta-analyses**

| Authors | Publication Year | Study Design | DES (%) | PCI (n) | CABG (n) | Region | Mean follow-up (months) |
| --- | --- | --- | --- | --- | --- | --- | --- |
| Capodanno et al. | 2012 | Prospective Registry | 100 | 84 | 118 | Europe | 12 |
| Conrotto et al. | 2014 | Prospective Registry | 100 | 218 | 86 | Europe | 36 |
| Ghenim et al. | 2009 | Retrospective Cohort (Propensity Adjusted) | 100 | 105 | 106 | Europe | 12 |
| Gomez et al.  (Mid GRC) | 2013 | Prospective Study | 89.3 | 58 | 117 | Europe | 36 |
| Gomez et al.  (High GRC) | 2013 | Prospective Study | 89.3 | 32 | 49 | Europe | 36 |
| Liu et al. | 2009 | Retrosepctive Cohort | 100 | 89 | 206 | Asia | 24 |
| Lu et al. | 2016 | Retrospective Study | 100 | 208 | 270 | Asia | 56 |
| Makikallio et al. | 2008 | Prospective Registry | 100 | 49 | 238 | Europe | 12 |
| Palmerini et al. | 2007 | Retrospective Study | 100 | 98 | 161 | Europe | 24 |
| Palmerini et al. | 2006 | Prospective Registry (the Bologna registry) | 60 | 157 | 154 | Europe | 24 |
| Rittger et al. | 2011 | Prospective Registry | 100 | 39 | 37 | Europe | 12 |
| Rodes-Cabau et al. | 2008 | Retrospective Study | 48 | 104 | 145 | Canada | 23 |
| Schampaert et al. | 2017 | RCT (EXCEL trial) | 100 | 167 | 159 | USA | 36 |
| Shimizu et al. | 2010 | Retrospective Study | 100 | 64 | 89 | Asia | 36 |
| Shiomi et al | 2015 | Prospective Registry | 75.83 | 364 | 640 | Asia | 60 |
| Wei et al. | 2016 | Prospective Study | 100 | 64 | 62 | Asia | 15 |
| White et al. | 2008 | Retrospective Study (Propensity Adjusted) | 100 | 120 | 223 | USA | 30 |

CABG = Coronary Artery Bypass Graft; DES = Drug Eluting Stent; PCI = Percutaneous Coronary Intervention; RCT = Randomized Controlled Trial

**Supplementary Table 2.** Newcastle-Ottawa Scale to assess the quality of each selected study.

| Studies | Selection | Comparability | Outcome |
| --- | --- | --- | --- |
| Capodanno et al. | ★★★★ | ★★ | ★★ |
| Conrotto et al. | ★★★★ | ★★ | ★★★ |
| Ghenim et al. | ★★★★ | ★★ | ★★★ |
| Gomez et al.  (Mid GRC) | ★★★★ | ★ | ★★★ |
| Gomez et al.  (High GRC) | ★★★★ | ★ | ★★★ |
| Liu et al. | ★★★★ | ★★ | ★★ |
| Lu et al. | ★★★★ | ★★ | ★★★ |
| Makikallio et al. | ★★★★ | ★ | ★★ |
| Palmerini et al. | ★★★★ | ★★ | ★★★ |
| Palmerini et al. | ★★★★ | ★★ | ★★★ |
| Rittger et al. | ★★★★ | ★★ | ★★ |
| Rodes-Cabau et al. | ★★★★ | ★★ | ★★★ |
| Shimizu et al. | ★★★★ |  | ★★★ |
| Shiomi et al | ★★★★ | ★★ | ★★★ |
| Wei et al. | ★★★★ | ★★ | ★★★ |
| White et al. | ★★★★ | ★★ | ★★★ |

**Supplementary Figure 1. Funnel-plot of all-cause mortality for analysis of publication bias – Symmetric inverted funnel indicates that publication bias is less likely.**

**
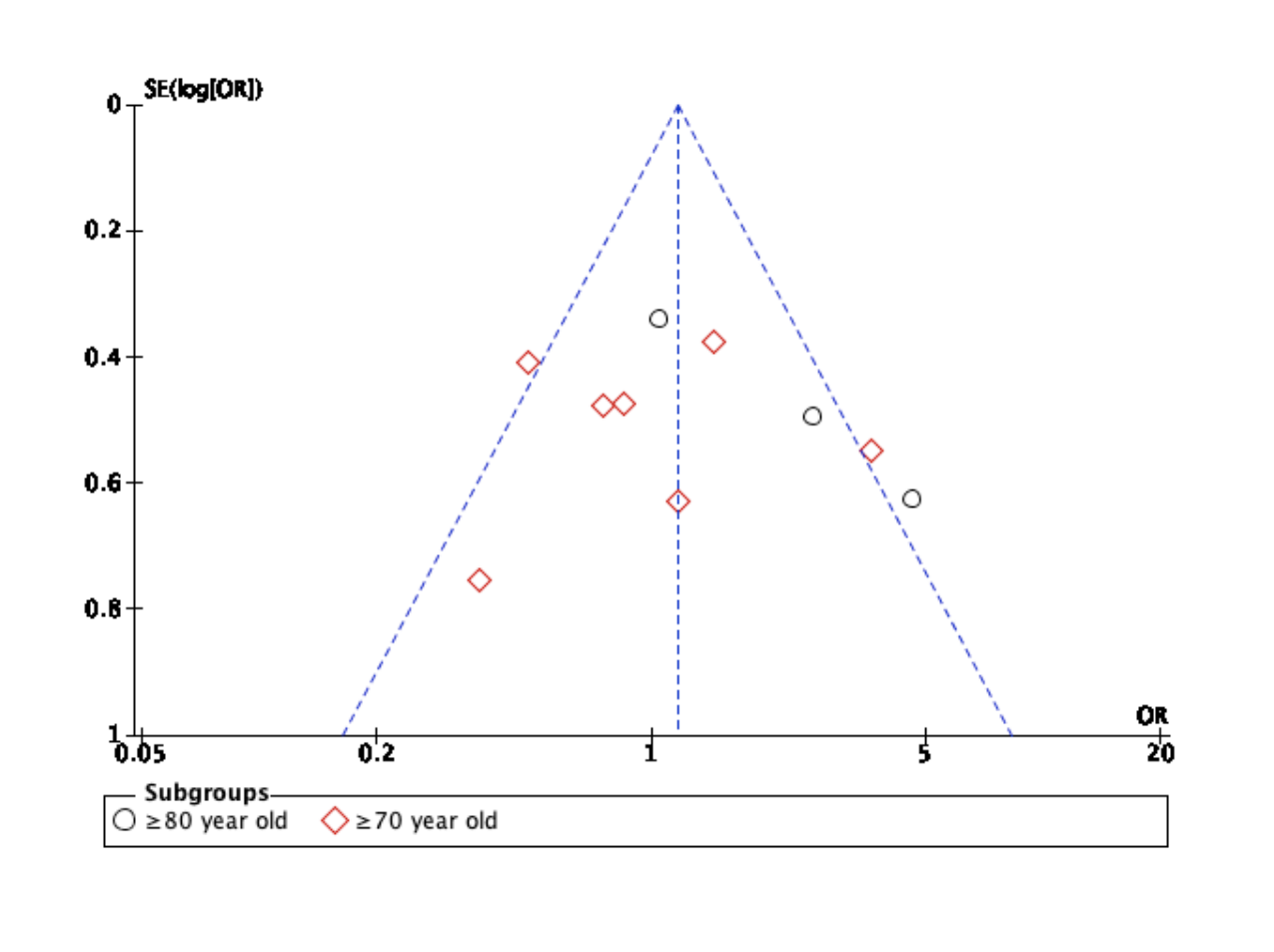
**

**Supplementary Figure 2. Forest plot of Stroke at (A) 30-days and (B) ≥36 months follow-up – Decreased 30-day stroke rate with PCI in ≥80-year-old patients and no statistically significant difference between PCI and CABG seen at ≥36 months.**


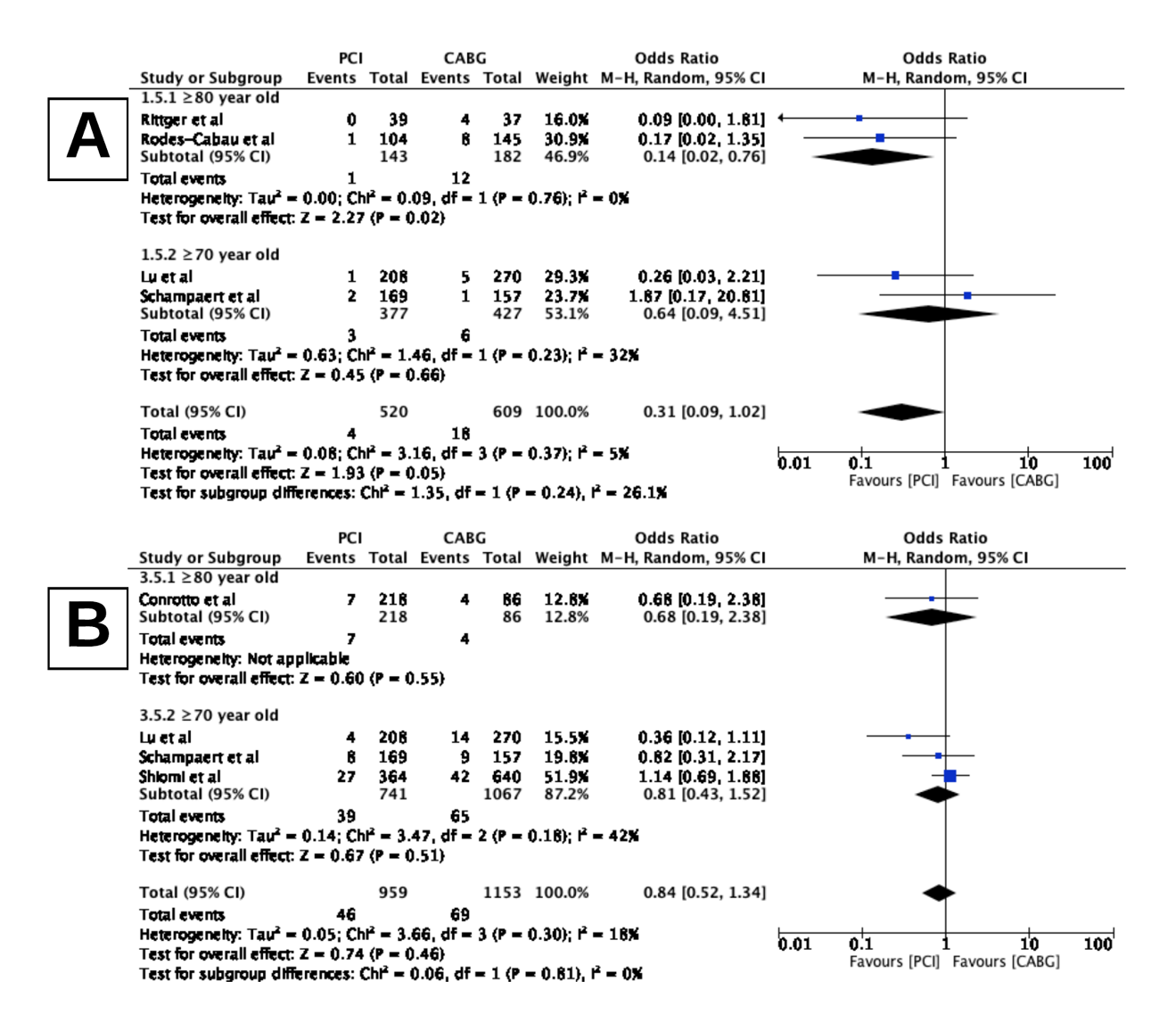

Supplement: Supplementary file 1 — Figure S1. Funnel‐plot of all‐cause mortality for analysis of publication bias—Symmetric inverted funnel indicates that publication bias is less likely. Figure S2. Forest plot of stroke at (A) 30‐days and (B) ≥36 months follow‐up—Decreased 30‐day stroke rate with PCI in ≥80‐year‐old patients and no statistically significant difference between PCI and CABG seen at ≥36 months. Table S1. Summary of all studies included in the meta‐analyses. Table S2. Newcastle‐Ottawa Scale to assess the quality of each selected study. [file CLC-42-1071-s001.docx]
